# The Relationship between Sleep Duration and Perceived Stress: Findings from the 2017 Community Health Survey in Korea

**DOI:** 10.3390/ijerph16173208

**Published:** 2019-09-03

**Authors:** Hwi Jun Kim, So Yeon Oh, Jae Hong Joo, Dong-Woo Choi, Eun-Cheol Park

**Affiliations:** 1 Department of Public Health, Graduate School, Yonsei University, Seoul 03722, Korea (H.J.K.) (S.Y.O) (J.H.J.) (D.-W.C.); 2 Institute of Health Services Research, Yonsei University, Seoul 03722, Korea; 3 Department of Preventive Medicine, Yonsei University College of Medicine, Seoul 03722, Korea

**Keywords:** community health survey, sleep duration, stress, national sleep foundation

## Abstract

Sleep is exceedingly important for our physical, physiological, psychological, and social health. Currently, few Koreans get the recommended daily amount of sleep. Stress can also have a major impact on our physiological, neurological, and mental health. In this study, we explored the correlation between sleep duration and perceived stress. The study used data from the Community Health Survey (CHS), 2017, which included 133,444 responses from Koreans. Sleeping time and stress were measured by self-diagnosis. The relationship between sleeping time and stress was analyzed using the chi-square test and multivariable regression. Both men and women felt the most stress when they slept for an average of 6 h a day. The results of the subgroup analysis showed that even when they sleep for the same time, younger people felt more stressed than older people. In the group that slept for an average of 6 h a day, women were the most stressed. We observed a correlation between sleeping time and stress in Korean adults. We found that about 16.7% of Koreans were sleeping for less than 5 h. This is less than the 7–9 h of sleep recommended by the National Sleep Foundation (NSF). In addition, stress was found to increase when sleep was insufficient. In particular, it was also observed that young people who slept for less than 8 h felt stressed more easily.

## 1. Introduction

Sleep has recently become a popular topic of research among academics. The results of this study show how sleep habits can affect physical, physiological, psychological, and social aspects [1,2,3]. Sleep duration seems particular important: excessively short or long sleep, compared to adequate amounts of sleep, is associated with a higher risk of mortality [4,5,6,7]{Kripke DF, 2002 #1}. In addition, sleep duration is also associated with the onset of various diseases such as depression, hypertension, diabetes, and obesity [8,9,10,11,12]. Both short and excessively long sleep duration (>10 h) appear to be associated with a risk of chronic disease [12,13]. According to a survey conducted by Gallup Korea in 2017, the average sleep duration in South Korea is about 6.5 h [14]. A survey by Statistics Korea, on the other hand, found an average sleep duration among Koreans of 7.7 h. These surveys suggested that many Koreans are not getting the recommended daily amount of 7–9 h of sleep suggested by the National Sleep Foundation (NSF). These averages are also substantially less than the average sleep duration of Organization for Economic Co-Operation and Development (OECD) countries, which is 8.3 h. However, only about 22.8% of Koreans reported being aware that they were not getting enough sleep [15]. 

Stress can be defined as a physiological reaction to protect our body from external stimuli. It can manifest as a state of physiological arousal and can have profound negative effects, particularly anxiety [16,17]. The autonomic nervous system and hypothalamic-pituitary-adrenal (HPA) axis are the two major bodily systems that govern stress [18,19]. Several experts have categorized stress into “good stress” and “bad stress,” or eustress and distress, respectively [20,21]. Both types of stress can directly or indirectly influence health. One study found a link between psychosocial stress and heart disease [22], while other studies have found that stress can affect the brain [23]. In the same survey that investigated sleep, 54.4% of the respondents felt generally stressed in their daily lives. This finding suggested that numerous people were exposed to stress. Interestingly, that same survey evaluated the prevalence of smoking and drinking and how stress related to attempts to quit these problematic behaviors. Among the respondents who smoked, 47.3% of them reported trying to quit smoking; however, over half of these respondents (52.6%) found that it was difficult to stop smoking because of stress. A similar finding was obtained for alcohol consumption; among those who tried to quit drinking or who tried to drink less, 34.7% said it was too difficult to do so because of stress. These findings suggest that stress can directly affect health, as well as indirectly influence it through the promotion of adverse health-related behavior. There are a number of studies on the correlation between sleep time and mental health worldwide. However, since the study of the relationship between sleep time and stress in Korea is very limited, we conducted this study to see if the same results would be obtained from Koreans. 

## 2. Materials and Methods 

### 2.1. Data Collection and Study Participants

For this study, we used data from the 2017 Community Health Survey, a cross-sectional, nationwide survey conducted by the Korea Centers for Disease Control and Prevention (KCDC). The total number of participants in this survey was 228,381. For this study, we excluded all individuals who did not respond to the question on stress (*n* = 104). We also excluded participants who did not respond to questions on age (*n* = 2452), marital status (*n* = 251), household income (*n* = 2006), occupational characteristics (*n* = 81,297), educational level (*n* = 106), region (*n* = 8364), smoking (*n* = 4), alcohol consumption (*n* = 4), depression (*n* = 18), suicidal ideation (*n* = 11), subjective health status (*n* = 6), physical activity (*n* = 53), unmet medical needs (*n* = 4), subjective body recognition (*n* = 16), and life satisfaction (*n* = 241). Thus, we analyzed the data of 133,444 participants (71,708 males and 61,736 females) as a representative sample. 

### 2.2. Variables

Our variable of interest was sleep time. The CHS asked “How much sleep do you have per day?”. The questionnaire was designed to make a response in hours and minutes. We classified these as ≤ 8, 7, 6, and <5 h for analysis.

Perceived stress was the dependent variable in this study. To measure it, respondents responded to the following question: “How stressful do you feel in your daily life?” Their response options were “feel very much,” “feel a lot,” “feel a little bit,” “hardly feel it,” and “do not feel it at all.” For the analysis, we categorized individuals who responded with “feel very much,” “feel a lot,” and “feel a little bit” as people who usually felt stressed in their daily lives, while those who responded with “hardly feel it” and “do not feel at all” were classified as people who did not usually feel stressed.

We also measured a number of sociodemographic, economic, health-related characteristics as covariates in the analysis. The sociodemographic characteristics included sex (male, female), age (20–29, 30–39, 40–49, 50–59, ≥60 years), marital status (married, unmarried, previously married (divorced, separated, widowed)), monthly household income (high, medium, medium-high, medium-low, low), occupation characteristics (white collar, pink collar, blue collar work), educational level (elementary school or below, middle school, high school, college, university, graduate school), and region (urban, rural). The health-related behaviors included smoking status (smoker: currently smokes cigarettes every day or occasionally; non-smoker: has never smoked or who has smoked in the past but does not smoke now) and alcohol status (regular alcohol drinkers: 1–16 times per month; non-regular drinkers: <1 time per month using the drinking frequency question for 1). In addition, the following variables were corrected with other covariates: depression (feel, do not feel), suicidal ideation (think suicide, do not think suicide), subjective health level (good, not bad, bad), physical activity(seldomly, 2–3 times, more than 4 times a week), unmet medical need (experienced, not experienced), subjective body recognition (thin, normal, obese), and life satisfaction (dissatisfied, satisfied).

### 2.3. Statistical Analysis

The chi square test and multiple logistics regression analysis were used to analyze the data. A *p*-value < 0.05 was considered to indicate a statistically significant result. The chi square test was used to examine the significant difference in stress depending on the sleep duration. Multiple logistic regression analysis was used to determine odds ratios (ORs) and 95% confidence intervals (CIs). Subgroup analysis was performed according to the sleep duration and stress. Statistical analyses were performed using SAS software, version 9.4 (SAS Institute, Cary, NC, USA).

## 3. Results

### 3.1. Study Participants

Table 1 shows the general characteristics of the study participants according to whether they felt stressed or not. Of the 71,708 male respondents, 56,976 (79.5%) were classified as people who usually felt stressed and 14,732 (20.5%) were classified as people who did not; of the 61,736 female respondents, 50,439 (81.7%) and 11,297 (18.3%) were categorized as “usually feeling stressed” and “not usually feeling stressed,” respectively. 

Among males, 9284 (71.3%) of the 13,019 individuals who slept for more than 8 h per day reported feeling stressed. Of the 24,166 participants who slept for 7 h, 19,446 (80.5%) reported feeling stressed. Of the 23,634 participants who slept for 6 h, 19,434 (82.2%) reported feeling stressed. Finally, among the 10,889 males who slept less than 6 h per day, 8812 (80.9%) indicated that they were feeling stressed. As for females, 8925 (76.0%) of the 11,737 who reported sleeping for more than 8 h usually felt stressed. Of the 20,260 females who slept for 7 h per day, 16,584 (81.9%) mentioned feeling stressed. In addition, 18,596 (84.4%) of the 18,596 females who slept for 6 h reported feeling stressed. Finally, among the 11,143 females who slept less than 6 h, 9228 (82.8%) reported feeling stressed. 

### 3.2. Factors that Predict Perceived Stress 

Table 2 shows the results of the logistic regression analysis with sleep duration and stress as the main independent and dependent variables, respectively. Both males and females showed increased odds of feeling stressed when the slept for less than 8 h per day. Specifically, males who slept for 7 h, 6 h, and less than 6 h had 1.38 (95% CI: 1.31–1.45), 1.42 (95% CI: 1.35–1.45), and 1.39 (95% CI: 1.30–1.48) times the odds of feeling stressed as did males who slept for 8 h or more, respectively. A similar trend was found among females: those who slept for 7 h, 6 h, and less than 6 h per day had 1.40 (95% CI: 1.32–1.48), 1.69 (95% CI: 1.59–1.80), and 1.33 times (CI: 1.52-1.75) the odds of feeling stressed as did females who slept for 8 h or more. A number of other variables were associated with stress, including age, occupational characteristics, education level, depression, suicidal ideation, unmet need medical, and life satisfaction. Thus, we performed a subgroup analysis with these variables.

### 3.3. Association between Sleep Duration and Stress Stratified by Age

**Figure 1:**
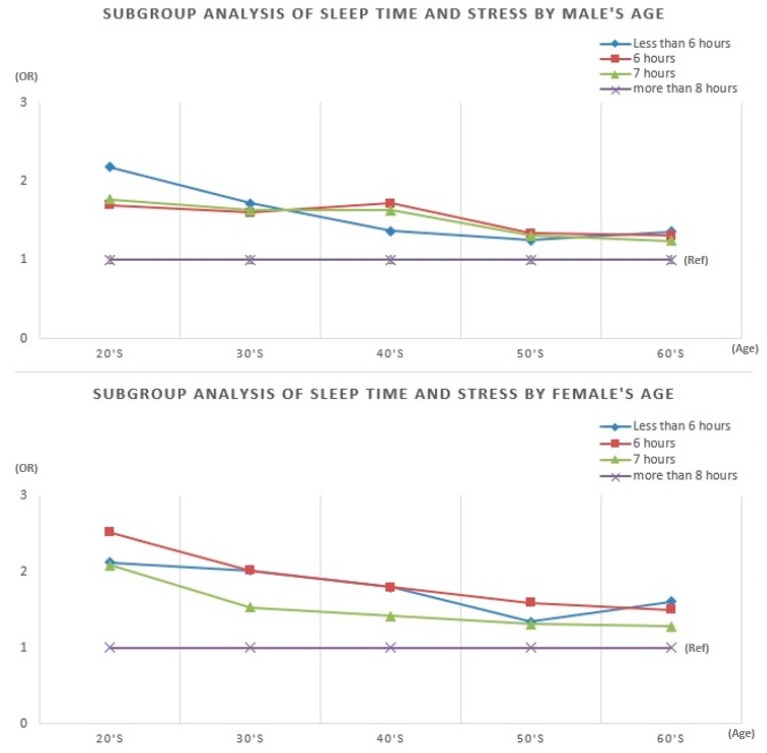
Subgroup analysis of sleep time and stress by age.

Figure 1 presents the results of the subgroup analysis by age. The results generally corresponded to those of the total sample: individuals who slept less than 8 h had higher odds of feeling stressed. We also found that, for each age group, the effect of sleep duration was stronger for females than for males. Among males in their 20s and 30s, the odds ratios were highest for those who slept less than 5 h. By contrast, among males in their 40s, the odds ratios were highest for those who reported sleeping for 6 h. The odds ratios did not differ much among those in their 50s and 60s. Among women, except for those in their 60s, the odds ratios were consistently highest for individuals who reported sleeping for 6 h. This phenomenon was somewhat different among females in their 20s and 50s, but were similar among those in their 30s, 40s, and 60s.

## 4. Discussion

This study was conducted to identify the relationship between daily sleep duration and perceived stress among adults over 20 years of age. There is currently much research on the relationship between sleep duration and aspects of mental health, such as depression and suicidal thoughts. Sleep is an essential element of everyday life that is closely related to health [24,25,26]. A recent hypothesis on the effects of sleep is that the brain adapts to our ever-changing environment through synaptic strengthening during wakefulness, while sleep helps to down-regulate this synaptic strengthening. Thus, insufficient sleep can adversely affect mental health [27]. In addition to mental health, sleep is exceedingly important for our physiology. For example, during sleep, cell growth and immune function are enhanced. Melatonin is the most active hormone secreted by humans during sleep, which plays an important role in controlling biorhythm. Melatonin also oxidizes cells to detoxify active oxygen, which causes aging, and engages in antitumor activities [28]. Sleep deprivation can promote the secretion of ghrelin and reduce leptin secretion, which are both associated with obesity. Ghrelin is a hormone secreted in the stomach that increases appetite—it is often called the “fasting hormone” because its levels increase rapidly during fasting and decrease after meals. A lack of sleep is known to increase ghrelin levels by up to 30%. Leptin is a hormone that acts in opposition to ghrelin by suppressing the appetite; people show reduced secretion of leptin when they are sleep deprived [29]. 

The present study showed a positive correlation between sleep duration and perceived stress among Korean adults. More specifically, people who sleep less than the NSF’s recommended daily amount of 7–9 h had higher odds of stress. In addition, we found that women had higher odds of stress than men for the same sleep durations. When examining the findings by age, younger participants also tended to experience higher odds of stress when their sleep was below the recommended daily amount. People who are stressed due to a lack of sleep appear to be more likely to commit suicide [30]. We hope that our findings will help to emphasize the need for publicity, education, and institutional improvement to ensure that Koreans have adequate sleep duration. 

Nevertheless, this study has some limitations. First, the cross-sectional design prevents us from inferring any causal relationships between sleep duration and stress. Second, the Community Health Survey evaluated average sleep duration per day based on the respondent’s memory. This likely led to recall bias. Third, we measured self-reported stress in this study. Experimental testing is necessary for a more accurate measurement of stress. Assessing stress levels with one question about stress may hardly reflect the actual stress levels. While it is possible to assess how stressed people feel subjectively, measurement bias or error can lead to inaccuracies in the relationship of stress to other subjective variables.

Despite these limitations, our study has several strengths. First, we used primary data suitable for Korean studies. These data are sufficient to confirm the average stress levels among Koreans because they were extracted from 133,444 questionnaires. Second, we confirmed that there is a correlation between sleep duration and stress, which suggests that publicity and education aimed at lowering stress among Koreans should focus on sleep. Furthermore, it calls for systemic environmental changes to ensure that Koreans can get enough sleep. 

## 5. Conclusions

We have found that almost half of all Koreans get less sleep than is recommended by the NSF. According to our findings, this might suggest that Koreans are frequently stressed. Furthermore, younger people tended to have higher odds of stress when they lacked sleep. Therefore, measures should be taken to ensure that individuals in their 20s and 30s get adequate sleep.

## Figures and Tables

**Table 1:** General characteristics of study observations (2017).

Variables			Male							Female					
	Total		Stress		Non-Stress		*p*-Value	Total		Stress		Non-Stress			*p*-Value
	*n*	(%)	*n*	(%)	*n*	(%)		*n*	(%)	*n*	(%)	*n*	(%)		
**Sleep time (hours)**							<0.0001							<0.0001	
8 ≤	13,019	18.2	9284	71.3	3735	28.7		11,737	19.0	8925	76.0	2812	24.0		
7	24,166	33.7	19,446	80.5	4720	19.5		20,260	32.8	16,584	81.9	3676	18.1		
6	23,634	33.0	19,434	82.2	4200	17.8		18,596	30.1	15,702	84.4	2894	15.6		
<6	10,889	15.2	8812	80.9	2077	19.1		11,143	18.1	9228	82.8	1915	17.2		
**Age (years)**							<0.0001							<0.0001	
20–29	5652	7.9	4844	85.7	808	14.3		6261	10.1	5711	91.2	550	8.8		
30–39	12,252	17.1	11,002	89.8	1250	10.2		8618	14.0	7837	90.9	781	9.1		
40–49	16,367	22.8	14,467	88.4	1900	11.6		13,663	22.1	11,932	87.3	1731	12.7		
50–59	17,364	24.2	13,895	80.0	3469	20.0		15,099	24.5	12,464	82.6	2635	17.5		
≧60	20,073	28.0	12,768	63.6	7305	36.4		18,095	29.3	12,495	69.1	5600	31.0		
**Marital status**							<0.0001							<0.0001	
Married	55,338	77.2	43,350	78.3	11,988	21.7		41,882	67.8	34,525	82.4	7357	17.6		
Once married (divorced, separated, bereavement)	4649	6.5	3541	76.2	1108	23.8		11,143	18.1	7989	71.7	3154	28.3		
Unmarried	11,721	16.4	10,085	86.0	1636	14.0		8,711	14.1	7925	91.0	786	9.0		
**Household income**							<0.0001							<0.0001	
Low	17,080	23.8	11,573	67.8	5507	32.2		19,845	32.1	14,623	73.7	5222	26.3		
Medium-Low	28,038	39.1	22,780	81.3	5258	18.8		19,811	32.1	16,683	84.2	3128	15.8		
Medium-High	16,415	22.9	14,061	85.7	2354	14.3		13,448	21.8	11,630	86.5	1818	13.5		
High	10,175	14.2	8,562	84.2	1613	15.9		8632	14.0	7503	86.9	1129	13.1		
**Occupational characteristics**							<0.0001							<0.0001	
White collar	21,508	30.0	18,659	86.8	2849	13.3		19,845	32.1	17,655	89.0	2190	11.0		
Pink collar	9560	13.3	8050	84.2	1510	15.8		16,906	27.4	14,308	84.6	2598	15.4		
Blue collar	40,640	56.7	30,267	74.5	10,373	25.5		24,985	40.5	18,476	74.0	6509	26.1		
**Educational level**							<0.0001							<0.0001	
Elementary school or less	8379	11.7	5090	60.8	3289	39.3		15,190	24.6	10,457	68.8	4733	31.2		
Middle school	7830	10.9	5440	69.5	2390	30.5		6880	11.1	5417	78.7	1463	21.3		
High school	23,681	33.0	19,093	80.6	4588	19.4		17,834	28.9	15,181	85.1	2653	14.9		
College	9251	12.9	8029	86.8	1222	13.2		7578	12.3	6740	88.9	838	11.1		
University	18,896	26.4	16,282	86.2	2614	13.8		12,223	19.8	10,860	88.9	1363	11.2		
Graduate school	3671	5.1	3042	82.9	629	17.1		2031	3.3	1784	87.8	247	12.2		
**Region**							<0.0001							<0.0001	
Urban	17,661	24.6	14,909	84.4	2752	15.6		14,579	23.6	12,676	87.0	1903	13.1		
Rural	54,047	75.4	42,067	77.8	11,980	22.2		47,157	76.4	37,763	80.1	9394	19.9		
**Smoking**							<0.0001							<0.0001	
Yes	27,856	38.9	23,338	83.8	4518	16.2		1886	3.1	1650	87.5	236	12.5		
No	43,852	61.2	33,638	76.7	10,214	23.3		59,850	97.0	48,789	81.5	11,061	18.5		
**Alcohol consumption**							<0.0001							<0.0001	
Yes	59,557	83.1	48,423	81.3	11,134	18.7		41,129	66.6	35,063	85.3	6066	14.8		
No	12,151	17.0	8553	70.4	3598	29.6		20,607	33.4	15,376	74.6	5231	25.4		
**Depression**							<0.0001							<0.0001	
Yes	2461	3.4	2344	95.3	117	4.8		3910	6.3	3727	95.3	183	4.7		
No	69,247	96.6	54,632	78.9	14,615	21.1		57,826	93.7	46,712	80.8	11,114	19.2		
**Suicidal ideation**							<0.0001							<0.0001	
Yes	3185	4.4	2961	93.0	224	7.0		4602	7.5	4345	94.4	257	5.6		
No	68,523	95.6	54,015	78.8	14,508	21.2		57,134	92.6	46,094	80.7	11,040	19.3		
**Subjective health status**							<0.0001							<0.0001	
Good	32,342	45.1	24,642	76.2	7700	23.8		22,778	36.9	17,921	78.7	4857	21.3		
Not bad	31,061	43.3	25,823	83.1	5238	16.9		28,618	46.4	24,180	84.5	4438	15.5		
Bad	8305	11.6	6511	78.4	1794	21.6		10,340	16.8	8338	80.6	2002	19.4		
**Physical activity**							<0.0001							<0.0001	
Seldomly	52,802	73.6	41,582	78.8	11,220	21.3		51,309	83.1	41,862	81.6	9447	18.4		
2–3 times	11,070	15.4	9193	83.0	1877	17.0		6182	10.0	5193	84.0	989	16.0		
More than 4 times a week	7836	10.9	6201	79.1	1635	20.9		4245	6.9	3384	79.7	861	20.3		
**Unmet medical need**							<0.0001							<0.0001	
Yes	6494	9.1	5807	89.4	687	10.6		8121	13.2	7327	90.2	794	9.8		
No	65,214	90.9	51,169	78.5	14,045	21.5		53,615	86.9	43,112	80.4	10,503	19.6		
**Subjective body recognition**							<0.0001							<0.0001	
Thin	13,126	18.3	10,415	79.4	2711	20.7		8580	13.9	6799	79.2	1781	20.8		
Normal	32,814	45.8	25,262	77.0	7552	23.0		27,385	44.4	22,023	80.4	5362	19.6		
Obese	25,768	35.9	21,299	82.7	4469	17.3		25,771	41.7	21,617	83.9	4154	16.1		
**Life satisfaction**							<0.0001							<0.0001	
Dissatisfaction	15,804	22.0	13,566	85.8	2238	14.2		15,132	24.5	13,393	88.5	1739	11.5		
Satisfaction	55,904	78.0	43,410	77.7	12,494	22.4		46,604	75.5	37,046	79.5	9558	20.5		
**Total**	71,708	100	56,976	79.5	14,732	20.5		61,736	100	50,439	81.7	11,297	18.3		

**Table 2:** Logistic regression analysis of the association between stress and sleep duration.

Variables	Male				Female			
	Adj.OR	95% CI			Adj.OR	95% CI		
**Sleep time (hours)**								
8 ≤	1.00	–		–	1.00	–		–
7	1.38	(1.31	–	1.45)	1.40	(1.32	–	1.48)
6	1.42	(1.35	–	1.50)	1.69	(1.59	–	1.80)
< 6	1.39	(1.30	–	1.48)	1.63	(1.52	–	1.75)
**Age (years)**								
20–29	3.04	(2.71	–	3.41)	2.99	(2.58	–	3.47)
30–39	3.69	(3.39	–	4.02)	3.01	(2.69	–	3.36)
40–49	3.09	(2.89	–	3.32)	2.12	(1.94	–	2.32)
50–59	1.74	(1.65	–	1.84)	1.59	(1.49	–	1.71)
≧ 60	1.00	–		–	1.00	–		–
**Marital status**								
Married	1.20	(1.11	–	1.30)	1.00	(0.89	–	1.12)
Once married (divorced, separated, bereavement)	1.04	(0.93	–	1.15)	0.67	(0.59	–	0.75)
Unmarried	1.00	–		–	1.00	–		–
**Household income**								
Low	0.73	(0.68	–	0.79)	0.88	(0.81	–	0.96)
Medium–Low	0.94	(0.88	–	1.01)	1.04	(0.96	–	1.13)
Medium–High	1.10	(1.02	–	1.18)	0.99	(0.91	–	1.07)
High	1.00	–		–	1.00	–		–
**Occupational characteristics**								
White collar	1.44	(1.35	–	1.52)	1.45	(1.34	–	1.58)
Pink collar	1.28	(1.20	–	1.37)	1.34	(1.27	–	1.43)
Blue collar	1.00	–		–	1.00	–		–
**Educational level**								
Elementary school or less	1.00	–		–	1.00	–		–
Middle school	1.29	(1.20	–	1.38)	1.35	(1.25	–	1.45)
High school	1.45	(1.36	–	1.55)	1.44	(1.33	–	1.56)
College	1.56	(1.42	–	1.72)	1.51	(1.35	–	1.69)
University	1.52	(1.40	–	1.65)	1.48	(1.33	–	1.65)
Graduate school	1.26	(1.12	–	1.42)	1.46	(1.23	–	1.72)
**Region**								
Urban	1.13	(1.08	–	1.19)	1.15	(1.09	–	1.22)
Rural	1.00	–		–	1.00	–		–
**Smoking**								
Yes	1.15	(1.10	–	1.20)	1.02	(0.87	–	1.18)
No	1.00	–		–	1.00	–		–
**Alcohol consumption**								
Yes	1.20	(1.14	–	1.26)	1.30	(1.24	–	1.37)
No	1.00	–		–	1.00	–		–
**Depression**								
Yes	3.11	(2.55	–	3.79)	2.77	(2.36	–	3.24)
No	1.00	–		–	1.00	–		–
**Suicidal ideation**								
Yes	2.82	(2.44	–	3.27)	2.93	(2.56	–	3.36)
No	1.00	–		–	1.00	–		–
**Subjective health status**								
Good	1.00	–		–	1.00	–		–
Not bad	1.76	(1.68	–	1.84)	1.80	(1.71	–	1.89)
Bad	1.87	(1.75	–	2.00)	2.14	(2.00	–	2.30)
**Physical activity**								
Seldomly	1.00	–		–	1.00	–		–
2–3 times	0.98	(0.92	–	1.04)	1.05	(0.96	–	1.14)
More than 4 times a week	1.08	(1.00	–	1.17)	1.06	(0.95	–	1.19)
**Unmet medical need**								
Yes	1.65	(1.52	–	1.80)	1.68	(1.55	–	1.82)
No	1.00	–		–	1.00	–		–
**Subjective body recognition**								
Thin	1.09	(1.03	–	1.15)	1.00	(0.94	–	1.07)
Normal	0.97	(0.92	–	1.01)	0.95	(0.90	–	0.99)
Obese	1.00	–		–	1.00	–		–
**Life satisfaction**								
Dissatisfaction	1.99	(1.88	–	2.10)	2.25	(2.12	–	2.38)
Satisfaction	1.00	–		–	1.00	–		–
